# Liquid biopsy—from bench to bedside

**DOI:** 10.1093/noajnl/vdac037

**Published:** 2022-11-11

**Authors:** Amitava Ray, Tarang K Vohra

**Affiliations:** Senior Consultant Neurosurgeon, Department of Neurosciences, Apollo Health City and Apollo Secunderabad, Hyderabad 500089, Telangana, India; Consultant Neurosurgeon, Department of Neurosciences, Apollo Health City, Hyderabad 500089, Telangana, India

**Keywords:** brain tumors, liquid biopsy

## Abstract

Over the last decade, molecular markers have become an integral part in the management of Central Nervous System (CNS) tumors. Somatic mutations that identify and prognosticate tumors are also detected in the bio-fluids especially the serum and CSF; the sampling of which is known as liquid biopsy (LB). These tumor-derived biomarkers include plasma circulating tumor cells (CTCs), cell-free DNA (cf/ctDNAs), circulating cell-free microRNAs (cfmiRNAs), circulating extracellular vesicles, or exosomes (EVs), proteins, and tumor educated platelets. Established in the management of other malignancies, liquid biopsy is becoming an important tool in the management of CNS tumors as well. This review presents a snapshot of the current state of LB research its potential and the possible pitfalls.

The holy grail of targeted therapy has been the promise of a system designed around a low-risk procedure that allows “real-time” targeting of tumors, sensitive enough to identify most pathological mutations, even those missed in a stereotactic biopsy.^1^ To this end, sampling of biological fluids like saliva, urine, CSF, pleural or even peritoneal fluid, which provide a rich source of circulating tumor cells, cell-free DNA (cfDNA)/(ctDNA), extracellular vesicles (EVs), proteins, and microRNA may prove to be a near-perfect fit.

The Evaluation of Genomic Applications in Practice and Prevention (EGAPP) initiative had recommended analytical validity, clinical validity, and clinical utility essential in adoption of liquid biopsies in clinical care.^2^ But, several regulatory authorities have approved of tests that do not meet all the criteria- the Cell Search System (Menarini Silicon Biosystems) is a Food and Drug Administration (FDA) approved CTC based test that accurately predicts prognosis of breast and colorectal cancer patients^3^ but fails to improve outcome. However, several tests like the cobas *EGFR* Mutation Test 2 (Roche) that detect *EGFR* mutations in Non-Small Cell Lung Cancer (NSCLC), the Epi pro Colon (Epigenomics AG) for colorectal cancer patients,^4^ the Guardant 360 CDx comprehensive genomic profiling (CGP) for the simultaneous assessment of single nucleotide variations (SNVs) or insertion and deletions in 55 tumor-associated genes^5^ and the 324 gene F1 Liquid CDx for use in (NSCLC) or prostate cancer meet all three criteria.^6^

This review presents a summary of the analytes (with special reference to CTCs, cfDNA, and exosomes) in liquid biopsy as they apply to brain tumors, enumerates the challenges of adoption in clinical practice.

## Circulating Tumor Cells (CTCs)

Ashworth discovered the presence of tumor cells in the circulation of a cancer patient- that now referred to as Circulating Tumor Cells (CTCs). These cells had been initially assessed as nonleucocytic and implicated in the development of distant metastases.^7^ CSF CTCs have been implicated in the prognostication of medulloblastomas for many years,^8^ but the exact role of CSF CTCs in the management of other primary and secondary malignant tumors remain much less clear. Though the specificity and sensitivity of CSF CTCs are much greater than that in the serum,^9,10^ the absolute contraindication in performing CSF draining procedures in patients with cranial tumors has severely limited its use. CTCs found in the serum are also known to form clusters with fibroblasts, parent tumor cells, endothelial cells, and platelets to protect against oxidative and immune stress that confer a survival advantage when compared to solitary cells.^11^ To increase the specificity and sensitivity of detection, these cells are captured using antibody‐based isolation techniques that have targeted specific cell surface proteins (EpCAM). However, this approach fails to detect the heterogeneity of surface proteins that is present on the CTCs^12^ or detect CTCs where EpCAMS are either absent or downregulated as in glial tumors.^13^ The frequency of release of CTCs into the bloodstream has not yet been established and it may not be ubiquitous throughout therapy.^12^ Negative selection such as erythrocyte lysis and density gradient separation, novel integrated approaches using subtraction enrichment and immunostaining along with fluorescent in situ hybridization and microfluidic systems using the dielectric properties of these malignant cells are now being used for CTC isolation.^14,15^ Though the role of CTCs in brain tumors is still being defined, CTCs have been extensively studied in breast cancer‐ its role in prognosis, and functions of clonal subtypes including roles of EpCAM positive and negative cells have been very well validated.^16^ Muller, *et al.* identified CTCs in the blood of glioma patients using Glial Fibrillary Acidic Protein (GFAP) as a marker in 29 out of 141 patients. CTCs detected had shared mutations with the tumor, confirming their tumor origins.^17^ Sullivan *et al.* used a micro‐fluidic system to deplete the circulating hemopoietic cells with antibodies captured CTCs in 13 out of 33 patients- CTCs exhibiting more mesenchymal than neural differentiation, suggesting a more aggressive origin.^18^ Gao *et al*. detected circulating tumor cells in 77% of glioma patients using selective enrichment and Fluorescent In Situ Hybridization (FISH),^19^ while Macarthur described the isolation of CTCs based on increased telomerase using an adenoviral detection system.^20^In a more recent article, Bang Christiansen *et al.* used a recombinant malarial protein rVAR2, to detect glioma cell lines by binding to cancer-specific oncofetal chondroitin sulfate (ofCS).^21^ In spite of this promise, the fact that CTCs are rare in brain tumors, have no specific cell surface marker, are difficult to harness, and require almost immediate processing do present barriers in its adoption. However, with the advent of single cell transcriptomics, live CTCs may provide vital additional information that aids the management of brain tumors in the future (Table 1).^6^

**Table 1.** A Summary Techniques and Results of CTCs in Brain Tumors

Marker (referencenumber)	Source	Glial tumor grades of glioma patients in study population if specified	Control population size	Biomarker detection methodology	sensitivity (SE) and specificity (SP)
^17^Müller C et al (2014)	Whole blood	Grade 4: 141	23	Fluorescence immunocyto-chemistry	SE: 21%; SP: 100%
^18^Sullivan JP et al (2014)	Whole blood	Grade 4: 33	6	STEAM immunofluorescence	SE: 39%; SP: 100%
^20^MacArthur KM et al (2014)	Whole blood	Grade 2-4: 11	30	Telomerase promoter-based assay	SE:72%;SP: 100%
^19^Gao F etal (2016)	Whole blood	Grade 2: 11; Grade 3: 9; Grade 4: 11	10	Subtraction enrichment and immunofluorescence	SE: 77%;SP: 100%
^22^Zhang W et al (2016)	Whole blood	Grade 2:11; Grade 3: 9; Grade 4: 12	178	Immunofluorescence	SE: 59%; SP: 100%
^23^Krol I et al(2018)	Whole blood	Grade 4: 13	3	Immunostaining	SE: 54%; SP: 100%
^21^Bang-Christensen SR et al (2019)	Whole blood	Glioma: 10	1	Immunoprecipitation	SE: 80%; SP:100%

Legend: *STEAM (SOX2, Tubulin beta-3, EGFR, A2B5, and c-MET)

## Circulating Tumor DNA (cfDNA/ctDNA)

cfDNA are cell-derived fragmented DNA usually 170–340 base pairs in length that are found in serum, CSF, and other body fluids, probably from dead and dying cells. ctDNA refers to those released exclusively from dead and dying tumor cells, but detection of ctDNA of active clones suggest active release by viable tumor cells as well.^24^ cfDNAs are not exclusively present in patients with tumors, but there seem to be an increase in the amount of ctDNA with an increase in the tumor size,^25^ the increase being possibly caused by an exhaustion of macrophage phagocytosis.^26^ In spite of low sensitivities^27^ caused by variable release and a relatively short half-life, the promise of sequencing the entire tumor genome piece meal, attracts interest.

The presence of ctDNA in the serum was first reported by Mandell and Mathias in 1948, but it was only in the 1970s that *KRAS* mutations were demonstrated in a patient with pancreatic cancer.^28^ The presence of ctDNA in gliomas was first demonstrated by Lavon and then by Marchrzak-Celinska. In the study of 70 tumors, Lavon *et al*. were able to demonstrate loss of heterozygosity of 1p19q or methylation of *MGMT* or *PTEN* in 62 out of 70 samples with a sensitivity of 55% for methylation and 51% for LOH and a specificity of 100%.^29^ Majchrzak-Celinska *et al.* also successfully detected methylation of *MGMT, RSSFIA, P14ARF,* and *P15INK4B* in the serum with similar specificity and sensitivity.^30^ IDH mutations were detected with 100% specificity and variable sensitivity in 80 glioma patients using COld PCR.^31^ Using the Gardent360 cell-free DNA detection assay, Piccioni *et al*. showed more than one somatic mutation in more than 50% of glioblastoma (GBM) patients.^32^ In a recently published study, Nassiri *et al*., using cell‐free methylated DNA immunoprecipitation and high‐throughput sequencing (cfMeDIP‐seq), demonstrated that methylation profiling of ctDNA could detect and discriminate different intracranial tumors, with similar cells of origin.^33^Studying the methylation of *Alu* elements in ctDNA of glioma patients, Chen et al. showed significant hypomethylation in the higher grade gliomas when compared to healthy controls, but no significant difference between the tumor grades or types of tumors.^34^Using targeted sequencing on a 50 gene chip in 27 high-grade gliomas Ahmed *et al.* found at least one mutation in *EGFR, VHL, KIT, PTEN, TP53* and *PIK3CA* genes, a possible low-cost solution for the developing world.^35^

The specificity and sensitivity of ctDNA in CSF has been shown to be greater than that of serum in multiple studies. De‐Mattos Arruda also demonstrated the presence of mutations of *EGFR, PTEN, ESR1, IDH1, ERBB2* and *FGRR2* in CSF of 12 glioma patients.^36^Huang *et al.* isolated ctDNA from patients with Diffuse Intrinsic Pontine Glioma (DIPG) and was able to detect *H3K27M* mutations in 4 of 5 patients.^37^ Izqueierdo *et al*. using digital droplet PCR detected *H3F3A_K27M* and *BRAF_V600E* mutations in DIPG and hemispheric pediatric gliomas.^38^ Pan *et al*. showed that mutations detected in ctDNA not were not in the tumor biopsy- but neither the ctDNA nor the stereotactic biopsy possessed absolute specificity and sensitivity.^39^

The value of liquid biopsies in the assessment of tumor response was demonstrated by Panditharatna *et al*.^40^ in a series of 110 liquid biopsies in 48 patients of pediatric DIPG, later corroborated by a similar study of 42 patients of adult GBMs by Bagley *et al*.^41^ MRI imaging correlated well with the ctDNA levels but CSF ctDNA was detected in 2 patients who previously did not have ctDNA in CSF prior to radiation- attributed to an increase in ctDNA release following cell damage. Increase in the CSF ctDNA with disease progression, reaching the maximum levels near-death was also demonstrated.^40^ In a similar study Fontanilles *et al*. showed that there was significant increase in the amount of cfDNA in the serum in patients with progressive disease, though the two targeted *TERT* promoter mutations were not reliably detected.^42^ However, using a novel digital droplet PCR (ddPCR) Muralidharan *et al.* were able to detect *TERT* mutations with a sensitivity and specificity of 69% and 100% respectively.^43^ In a study of 130 terminal patients of GBM and metastatic brain tumors being treated with intra nasal perillyl acohol based therapy, Faria *et al.* showed that the amount of cfDNA was significantly higher in these patients when compared to the healthy controls and increased levels of cfDNA following cessation of therapy corroborated MRI findings (Table 2).^44^

**Table 2.** A Summary of Some of the Cell-Free DNA Studies Demonstrating the Methods Used and Their Sensitivity and Specificity

Marker (reference number)	Source	Grades of glioma patients in study population if specified	Control population size	Biomarker detection methodology	Marker AUC, accuracy or sensitivity (SE) and specificity (SP) if measured
^29^Lavon et al (2010)	Serum	Grade 2:14; Grade 3:27; Grade 4:29	20	salting-out method	SE: 51%; SP: 100%
^31^Boisselier et al (2012)	Plasma	Grade 2:28; Grade 3:42; Grade 4:10	30	Cold PCR	SE: 60%; SP: 100%
^45^Shi W et al (2012)	Serum/ CSF (ALU115 & ALU247)	Grade 1: 3; Grade 2: 35; Grade 3: 14; Grade 4: 18	22	qPCR	ALU 115: SE: 75; SP: 87.5% ALU 247: SE:85%; SP:87.5%
^30^Majchrzak-Celińska et al (2013)	Serum	Grade 2:2; Grade 3:6; Grade 4:9	16	Spectrophotometry	SE: 81%; SP: 97%
^46^Chen J et al (2013)	Serum (Alu methylation)	Grade 1,2: 32 Grade 3,4: 33	30	DNA sequencing	SE: 84.40%: SP:80.19%; AUC: 0.893
^47^Wang et al (2015)	CSF	Grade 1:4, Grade 2: 3, Grade 3: 2, Grade 4: 11, MB: 6; EM: 7		TS followed by WES	SE: 74%
^36^De Mattos Arruda et al (2015)	CSF	Grade 4: 4 Mets: 8		ddPCR/WES	SE: 100%
^44^Faria G et al (2018)	Serum	Grade 4: 122	130	Fluorimetry	NA
^34^Chen J et al (2016)	Serum (Alu methylation)	Grade 1,2: 38; Grade 3,4: 71	50	DNA sequencing	AUC: 0.86
^48^Martínez-Ricarte et al (2018)	CSF	Grade 2: 8; Grade 3: 2; Grade 4: 10		TS	SE: 85%
^49^Mouliere F et al (2018)	Plasma	Grade 4: 34	65	WES	AUC: 0.80–0.99
^50^Mouliere (2018)	CSF	Glioma: 13		WES	SE: 39%
^51^Miller (2019)	CSF	Glioma: 85		NGS	SE: 49.4%
^35^Ahmed KI et al (2019)	Serum	Grade 4: 27	0	TS	SE: 88.8% SP: 100%
^52^Zill (2019)	Plasma	Glioma: 107		NGS	SE: 51%
^53^Mueller et al (2019)	Plasma	DMG			SE: 92%
^39^Pan C (2019)	CSF	DMG: 57		ddPCR	SE: 97.3% SP: 83%
^41^Bagley SJ(2020)	Plasma	Grade 4: 42	42	qPCR	AUC: 0.99
^33^Nassiri F et al (2020)	Plasma (methylomes)	Glioma: 112	59	Illumina HumanBeadChip 850K array	AUC: 0.99
^54^Bagley (2021)	Plasma	Grade 4 (IDH wild type): 62		SYBR Green-based qPCR	SE: 55%
^38^Izquierdo et al(2021)	Plasma, CSF, tumor cyst	DMG: 44		ddPCR	
^55^Yu J et al (2021)	Tumor In situ Fluid	Grade 2: 8; Grade 3: 8; Grade 4: 14		TS	
^56^Sun Y et al (2021)	CSF	MB: 58		NGS	

Legend: CSF, Cerebro Spinal Fluid; COLD PCR, CO-amplification at Lower Denaturation temperature PCR; ddPCR, Digital Droplet PCR; DMG, Diffuse Midline Glioma; EM, Ependymoma; MB, Medulloblastoma; Mets, Metastasis; qPCR, Quantitative Polymerase Chain Reaction; NGS, Next Generation Sequencing; TS, Targeted Sequencing; WES, Whole Exome Sequencing.

## Exosomes

Exosomes are nano‐vesicles between 40–100 nm in diameter and are the end product of the recycling endosomal pathway.^57^ Though they were initially considered to be cellular waste,^58^it is now known that exosomes play important roles in angiogenesis, migration, proliferation of tumor cells^,59,60^ and in the maintenance of the immunosuppressive tumor environment.^61^ Exosomes consist of a lipid bilayer which contains both cytoskeletal and non‐membrane proteins suggesting a tissue-specific origin,^62^ as well as miRNAs, mRNAs, and either single or double‐stranded DNA,^63^ the DNA shown to contain the same mutations as the tumor.^63^

The appreciable amount of nucleic acids lend exosomes to biomarker investigations. In certain cases, the nucleic acid concentration in exosomes are several times that found in parent cells, suggesting active “packing”.^60^ Noerholm *et al.* analyzed serum‐derived exosomes from 10 GBM patients and found significantly lower levels of 4 ribosomal function genes when compared to normal‐ *RPL11, RPS12, TMSL3,* and *BM2*.^64^ Skog *et al.* showed EGFRvIII mRNA in detectable levels in GBM microvesicles.^60^ In a similar study of 88 patients of GBM, Manda *et al.* compared the presence of EGFRVIII mRNA in the tumor vs the exosome in patients with gliomas and showed that EGFRVIII mRNA can be detected in the exosomes with approximately 85% accuracy.^65^

Noncoding microRNAs (miRNAs) have also been widely investigated in the exosomal cargo- both in the serum and the CSF. Manterola *et al.* found increased levels of RNU6‐1 and miRNA‐320 and miRNA‐574‐3p that correlated with GBM diagnosis with a specificity and sensitivity of approximately 86%.^66^ Figueroa *et al.* showed EGFRVIIImRNA can be detected in the CSF in 81 patients with GBM, with a sensitivity and specificity of 60% and a specificity of 98% respectively.^67^ In a similar study of GBMs, Akers *et al.* demonstrated a 10‐fold increase in the miR‐21 levels when compared to controls.^68^ These results were then validated with a larger set of 29 patients yielding a diagnostic specificity and sensitivity of 87% and 93% respectively.^69^ Using unbiased high throughput next‐gen’ sequencing and an integrative bioinformatics platform, Ebrahimkhani *et al.* found 26 differentially expressed miRNAs in GBM patients when compared to healthy controls. The selected panel of seven miRNAs‐ predicted GBM diagnosis with a 91% accuracy. Within this multivariate model, four miRNAs ‐miRNA‐182, miR‐328‐3P, miR340‐5p, miR‐486‐5p distinguished GBMs from healthy controls with 100% accuracy.^58^ Using on‐chip immunofluorescence to measure the concentration of GFAP and TAU proteins in exosomes, Lewis *et al.* found it was possible to differentiate the plasma of controls from that of GBM patients with 60% and 94% sensitivity and specificity respectively. This was later validated using another independent cohort of patients.^70^ The protein content in exosomes in patients with GBM were seen to be differentially expressed from the normal^71^ and the EGFR protein was seen to be expressed in about 25% of glioma patients.^63^

Recently, exosomes have also been shown to actively induce the loss of tumor suppressors like pTEN in metastatic tumors.^22^ In addition, exosomes help regulate the adaptation of the tumor cells to the induced hypoxic environment by upregulating transcription of small nucleolar RNA, C/D box 116-21, and downregulating potassium voltage-gated channels.^72^ What has been of increasing therapeutic interest is the ability of exosomes to home-in on particular cells and modify their behavior by off-loading their nucleic acid cargo-which may be modified to carry drugs or vaccines. Exosomes derived from Natural Killer Cells have already been shown to exhibit anti-tumor properties, though the exact mechanism of such actions is unclear.^73^ When combined with modalities like Focused Ultrasound Therapy that transiently open the blood–brain barrier, exosomes may have a therapeutic role in brain tumors.^74^

## Other Methods

Using total reflection(ATR)- Fourier transformed Infrared Spectroscopy (FITR) alongside machine learning algorithms, Butler *et al*. were able to differentiate patients with brain cancer form the control population with a specificity and sensitivity exceeding 90%.^75^ Platelets also have been recently in the spotlight for their ability to transfer tumor-associated molecules using their RNA processing machinery. Analysis of such tumor “educated” platelets(TEPs) from 228 samples including GBMs differentiated cancer patients from healthy controls with an accuracy of 84-96%.^76^ Several plasma protein levels like interleukin 2 (IL-2) and its receptor, tumor necrosis factor alpha (TNFa), transforming growth factor beta (TGFb), chitinase-3-like protein 1 (CHI3L1, also known as YKL-40), neural cell adhesion molecule (NCAM), and neuropeptide Y (NPY) have been investigated as potential biomarkers in brain tumors.^77^

## From Bench to Bedside

The widespread adoption of liquid biopsy in clinical practice will require more than just clinical and analytical validation but proven clinical benefit. Though some of the problems with clonal proliferation have largely been answered, questions about the exact function of exosomes and the origin of cfDNA remain. The variability of outcomes will have to be reconciled by guidelines defining preanalytical conditions including blood collection, plasma preparation, and analyte extraction.^78^ Clinical trials need to be designed where liquid biopsy is used to fill a specific gap in the care pathway, complementary to radiological or other established investigations. While multi-analyte testing will improve specificity and sensitivity,^79^ the additional data does increase the complexity of interpretation, increasing cost and sometimes confounding results. For health services around the world grappling with the pandemic and its aftermath, even a small increase in cost may be more than a significant hurdle in its adoption.

## Conclusion

Despite the present-day challenges, the potential benefits of a tool that provides real-time assessment of patients cutting through the maze of heterogeneity is there for all to see. As global collaboration increases, it is only a matter of time before this tool plays a significant role in our fight against cancer.
